# Design and Mechanical Analysis of Bionic Foldable Beetle Wings

**DOI:** 10.1155/2018/1308465

**Published:** 2018-08-09

**Authors:** Caidong Wang, Chen Wang, Yu Ning, Lumin Chen, Xinjie Wang

**Affiliations:** ^1^College of Mechanical and Electrical Engineering, Zhengzhou University of Light Industry, Zhengzhou 450002, China; ^2^Henan Key Laboratory of Intelligent Manufacturing of Mechanical Equipment, Zhengzhou 450002, China

## Abstract

In order to improve the flight performance of collapsible aircrafts, a novel mechanism of bionic foldable wings of beetle is designed based on the four-plate mechanism theory. The folding and unfolding movements of the bionic foldable wings are driven by motor and torsion hinges. Based on the D-H method, a kinematic model of wings is established to analyze the dihedral angle of adjacent plates. The folding ratio of an area in different plate creasing angles has been derived and calculated. Utilizing the kinematic and static models produced, as well as considering the folding ratio and output motor torque, the optimal physical parameters of folding wings are obtained. Dynamic models of rigid and flexible wings were established using ADAMS, and a motion simulation was performed. The relationship between dihedral angle and torque during the folding process of both rigid and flexible wings was obtained. The results provide a better understanding of the folding mechanism through the formulation of rigid-flexible wing analysis, as well as demonstrating a novel design of insect-mimicking artificial wings for small aerial vehicles.

## 1. Introduction

Bionics is one of the most important examples of researchers seeking better inventions and engineering designs. The flying ability of birds, insects, and other creatures is amazing. The study of flight principles observed in nature can greatly improve the performance of existing aircrafts and promote the development of new and unique aircrafts [1–3]. Compared with the traditional aircrafts, flapping-wing air vehicles have advantages such as simpler design, lower noise, higher efficiency, and better environmental protection [4–6]. However, observation of insect flight is a relatively recent field of study. Beetles (Coleoptera) can drill into soil and water after storing their flexible wings under their sheath wings. The folding ratio of these flexible wings is relatively large [7]. At present, the bionic design of foldable wings is mainly concentrated structural considerations, which restricts the improvement of motion performance [8]. Therefore, it is important to study on the bionic design and the motion mechanism of flexible foldable wings.

The good flight characteristics of the foldable wings have attracted a significant number of researchers. Muhammad et al. [9] divided the membrane structure of the *Allomyrina dichotoma* beetle wings. The characteristics of folding and unfolding of wings were analyzed. Two types of artificial wings driven by a shape memory alloy with 5 V, 1.5 A power supply were developed. Truong et al. [10] used a double four-bar mechanism for the folding of artificial wings, but the change of angle between the two main lines of wings was driven manually. Based on uniform velocity rigidity model, Rui et al. [11] obtained a new model of flapping wings of variable velocity by adding the influence of the change of the flapping rate and the change of the wing shape. This flapping model more closely captures bird wing flexibility. Zhenjun et al. [12] used the Lagrange method to infer the coupling equation of rigid-elastic deformations of flexible aircraft. Cheng [13] studied the deformation characteristics of flying wings of dragonfly using a projected sinusoidal grid method. Ha et al. [14] successfully developed a method based on a minitensile test system and the DIC method to measure Young's modulus and Poisson's ratio of the membrane of the hind wing of the *Allomyrina dichotoma* beetle. While most studies consider wingbeat kinematics critical to lift generation, few address the shape and mechanical properties of the wings themselves [15, 16]. Recent discoveries in the field of flapping-wing aerodynamics have demonstrated that flexible wings can generate more lift than rigid wings. Ghommem et al. [16] used the unsteady vortex lattice method together with a gradient-based optimizer to obtain optimized wing shapes that give maximum efficiency. This study also found that the optimal wing shapes are highly dependent on reducing the wingbeat frequency. Tay [17] performed 3D simulations to determine the effects of prescribed deformation on different types of wings under various flapping configurations. Bluman and Kang [18] found that the flexible wings require 32%–94% less power than rigid wings.

Haas and Wootton and Haas and Beutel proposed the four-board model for the folding and unfolding of insect hind wings [19, 20] but did not explain how to achieve it through mechanisms. Based on the four-plate model theory proposed by Haas and Wootton and Haas and Beutel, the mechanism of bionic foldable flexible wings of the beetle is designed in this paper. The mechanism of the foldable wings is driven by a motor and torsion hinge. The dynamic model of the rigid and flexible wings is established using ADAMS, and a motion simulation of bionic foldable wings is performed.

## 2. The Design of the Mechanism of Foldable Wings

**Figure 1:**
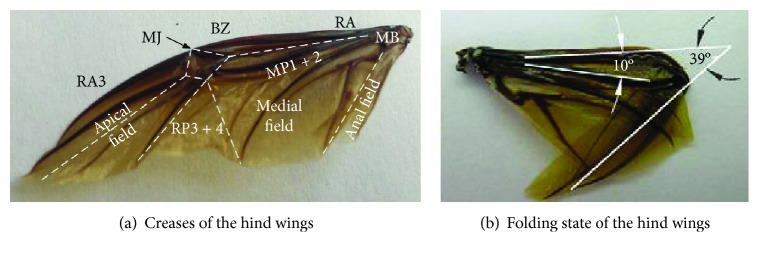
Hind wing shape analysis of *Allomyrina dichotoma* beetle.

The folding and unfolding configuration of the *Allomyrina dichotoma* beetle hind wings is shown in Figure 1. The hind wings are composed of the apical field, middle field, anal field, and wing veins. By observing the process of unfolding and folding of the unicorn hind wings, there are five creases in the folding process of the hind wings, as shown by the dotted line in Figure 1. Due to the area in the anal field that is smaller, its effect can be ignored. Then, the four creases of the hind wing intersect with one point.

**Figure 2:**
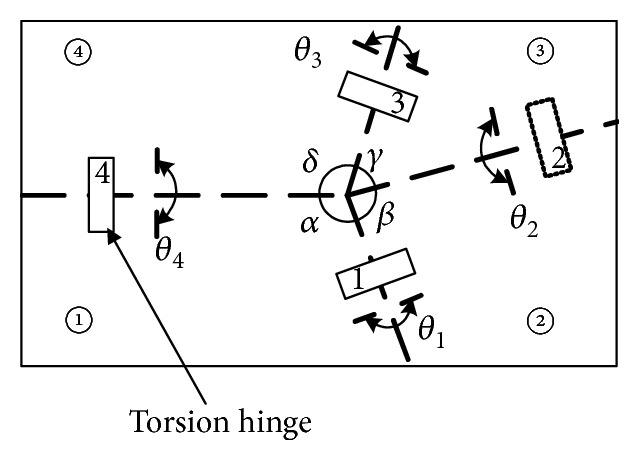
Schematic diagram of wings.

During the wing folding process, elastic energy is stored in resilin, a rubber-like substance [20]. Resilin can be found at some locations in a hind wing, such as medial bridge (MB), bending zone (BZ), and marginal joint (MJ). But it is very difficult to imitate the biological characteristics of resilin to drive the hind wings to achieve folding and unfolding motion. Through an analysis of the physical form and movement of the *Allomyrina dichotoma* beetle, combined with the theory of mechanics, a model of the bionic wings is established. The model is shown in Figure 2.

The mechanism of foldable wings consists of four plates with 1 degree of freedom. The adjacent wing plates are connected by torsion hinges, which are made of electroactive polymer (EAP) material. The folding of the hind wings is driven by the motor with elastic rope and unfolding by the elastic driving force of the electroactive polymer (EAP) torsion hinge. The creasing angle relationships for each plate of the wings are *δ* + *β* = *π* and *γ* + *α* = *π*. The angle of adjacent plates as the dihedral angle *θ* is shown in Figure 1. The wing plates are connected by torsion hinges. Plate ④ is connected to the aircraft body at the base. Plate ③ is active against the elastic force of the torsion hinges and is rotated toward plate ④ and is driven by the motor. Plates ① and ② are driven by the torsion hinges as followers. The principle of the foldable wings is that when the wings are folding, the motor drives the torsion hinges to bend and drives the wings to complete the folding movement. When the wings are unfolding, the wings are driven by the elastic potential of the torsion hinges themselves.

Folding performance is a key factor to consider when designing a folding wing mechanism. Under the constraint of satisfying the output motor torque, the folding ratio of wings is given priority. In general, the design of the folding mechanism of wings should satisfy the following principles:

1. The structure of wings should be simple, small in size, and lightweight.
2. The creasing angle of adjacent wing plates should be reasonably designed in order to meet the folding ratio and motor torque requirements.
3. The torsion hinges between the plates should be locked in the movement, thereby avoiding unwanted relative displacement of the plates.
4. In order to avoid coupling motion between the plates, the movement of folding and unfolding of wings should be continuous and smooth.

In order to ensure adequate transmission performance, it is necessary to reasonably design the size and angle of plates and avoid dead spots to prevent becoming stuck in the process of the movement. As such, the requirements *δ* > 90° and *γ* < 90° should be met.

The function of torsion hinges is to connect and fix the plates. In the process of flapping, the wings are in an expanded state and the bending moment, torque, and shear stress caused by the aerodynamic load on the airfoil are transmitted as concentrated force through the torsion hinges. At this moment, the folding wing mechanism only needs to withstand its own gravity and air resistance.

## 3. Characteristics of Foldable Wings

### 3.1. Analysis of Kinematics

**Figure 3:**
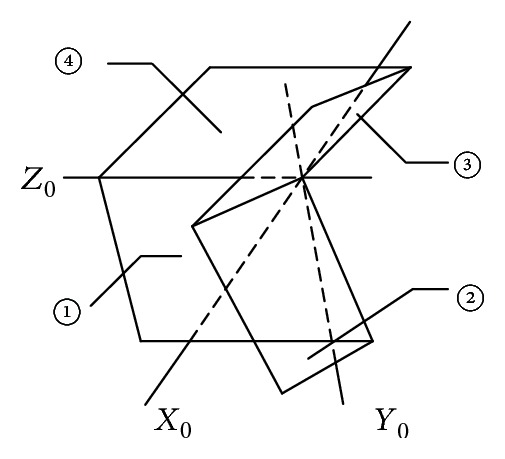
Schematic diagram of folding wings.

**Figure 4:**
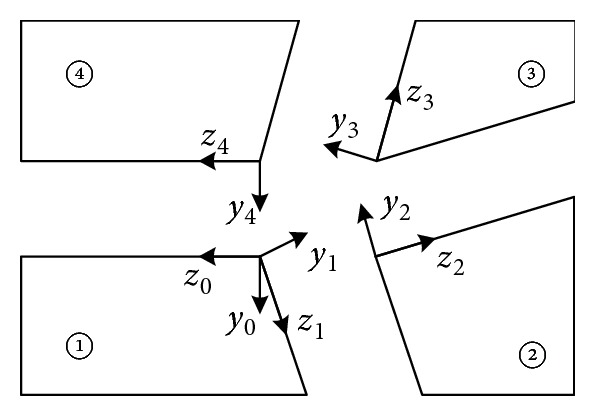
Simplified kinematics model.

The present simplified kinematic model of foldable wings is shown in Figure 3. The coordinate system of each plate is set up by the D-H parameter method, as shown in Figure 4. The coordinate system parameters of the foldable wings are shown in Table 1.

According to the kinematic homogeneous transform theory, the transformation matrix of adjacent plate ② is

(1) T12=cβsβcθ1−sβsθ10−sβcβcθ1−cβsθ100sθ1cθ100001,T41=cαsαcθ4−sαsθ40−sαcαcθ4−cαsθ400sθ4cθ400001,T32=cγ−sγ00sγcθ2cγcθ2−sθ20sγsθ2cγsθ2cθ200001,T43=cδ−sδ00sδcθ3cδcθ3sθ30−sδsθ3−cδsθ3cθ300001,

where *sθ*_*i*_ = sin*θ*_*i*_ and *cθ*_*i*_ = cos*θ*_*i*_.

From the space position constraint of plate ②, we can get _1_^2^**T**_4_^1^**T** = _4_^2^**T** = _3_^2^**T**_4_^3^**T**.

(2) A=T42=T12T41=cαcβ−sαsβcθ1cθ4cβsα+cαsβcθ1−sβsθ1sθ4−sθ4cβsα+cαsβcθ1−sβcθ4sθ10−cαsβ−cβsαcθ1−cθ4sαsβ−cαcβcθ1−cβsθ1sθ4sθ4sαsβ−cαcβcθ1−cβcθ4sθ10−sαsθ1cθ1sθ4+cαcθ4sθ1cθ1cθ4−cαsθ1sθ400001,B=T42=T32T43=cδcγ−sδsγcθ3−cγsδ−cδsγcθ3−sγsθ30sδsθ2sθ3+cγcθ2cθ3+cδsγcθ2cδsθ2sθ3+cγcθ2cθ3−sδsγcθ2cγcθ2sθ3−cθ3sθ20cδsγsθ2−sδcθ2sθ3−cγcθ3sθ2−cδcθ2sθ3−cγcθ3sθ2−sδsγsθ2cθ2cθ3+cγsθ2sθ300001.

Due to the homogeneous transformation matrix **A** = **B**, it is obtained that the relations for *θ*_1_, *θ*_2_, *θ*_3_, and *θ*_4_ are

(3) $$\begin{array}{c} \theta_{1}=\theta_{3},\\ \theta_{2}=\theta_{4}=\frac{c\gamma s\delta +c\theta_{3}s\gamma c\delta }{s\delta s\theta_{3}+\left(s\gamma c\delta +c\theta_{3}c\gamma s\delta \right)\left(s\gamma s\delta -c\theta_{3}c\gamma c\delta +c\theta_{3}\right)/\left(s\theta_{3}c\gamma -s\theta_{3}c\delta \right)}. \end{array}$$

**Figure 5:**
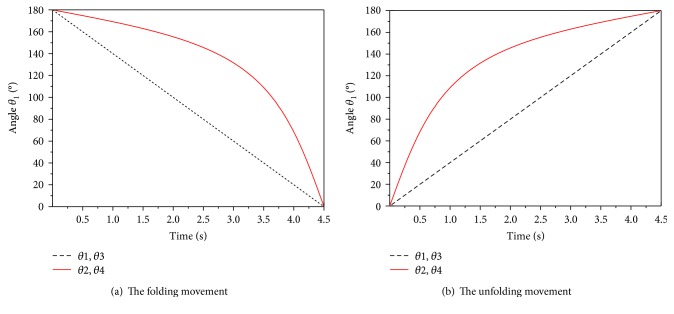
The dihedral angle in the folding/unfolding movement.

The kinematic model of foldable wings was programmed using MATLAB software. The simulation results of the folding and unfolding movement of wings are shown in Figure 5. When angle *θ*_1_ moves along the desired trajectory, the curve of angle *θ*_2_ can be obtained according to the above mathematical model. In the movement of folding, the motion of *θ*_2_ is smooth between 180 ° and 130 °. The change of angle speeds up between 130° and 0°. That is, the trend of the change in angle in the folding process is to be slow and then fast. In the movement of unfolding, the change of *θ*_2_ is faster between 0° and 50°, while the change in angle is slower between 50°–180°. Therefore, the tendency of the angle curve in the unfolding process is to be fast and then slow.

### 3.2. Mechanical Modeling and Analysis

In the movement of folding and unfolding of wings, the torsion hinge between plate ③ and plate ④ is driven by the motor to fold the wings. *OC* is selected as the rotation axis for torque analysis. The output torque of the motor is affected by the posture of wings and the gravitational forces of the plates. In fact, the movement of plate ① lags behind plate ③, which can be expressed using the dihedral angle *θ*_1_ > *θ*_3_. The output torque of the motor is given by *M*; the torque of plates acting about the axis of rotation is *M*_1_, *M*_2_, and *M*_3_. The bending deformation stress of the torsion hinges is given by *f*_1_, *f*_2_, *f*_3_, and *f*_4_, where *f*_1_ = *f*_3_ and *f*_2_ = *f*_4_. The torque of the torsion hinges acting about the axis of rotation are *Mf*_1_, *Mf*_2_, *Mf*_3_, and *Mf*_4_. The total torque is Σ*M*_*F*_ and the total resistance torque is Σ*M*_*f*_.

The equilibrium equations for the static analysis of the folding wing movement are as follows:

1. When the angle *π*/2 < *θ*_3_ < *π*, the total resistance torque is Σ*M*_*f*1_ = *M*_1_ + *M*_2_ + *M*_3_ + *Mf*_1_ + *Mf*_2_ + *Mf*_3_ + *Mf*_4_, the total torque is Σ*M*_*F*1_ = *M*, and the equilibrium equation is Σ*M*_*F*1_ = Σ*M*_*f*1_.
2. When the angle *θ*_3_ < *π*/2 and *θ*_1_ > *π*/2, the total resistance torque is Σ*M*_*f*2_ = *M*_1_ + *Mf*_1_ + *Mf*_2_ + *Mf*_3_ + *Mf*_4_, the total torque is Σ*M*_*F*2_ = *M*_2_ + *M*_3_ + *M*, and the equilibrium equation is Σ*M*_*F*2_ = Σ*M*_*f*2_.
3. When the angle *θ*_3_ < *π*/2, the total resistance torque is Σ*M*_*f*3_ = *Mf*_1_ + *Mf*_2_ + *Mf*_3_ + *Mf*_4_, the total torque is Σ*M*_*F*3_ = *M*_1_ + *M*_2_ + *M*_3_ + *M*, and the equilibrium equation is Σ*M*_*F*3_ = Σ*M*_*f*3_.

**Figure 6:**
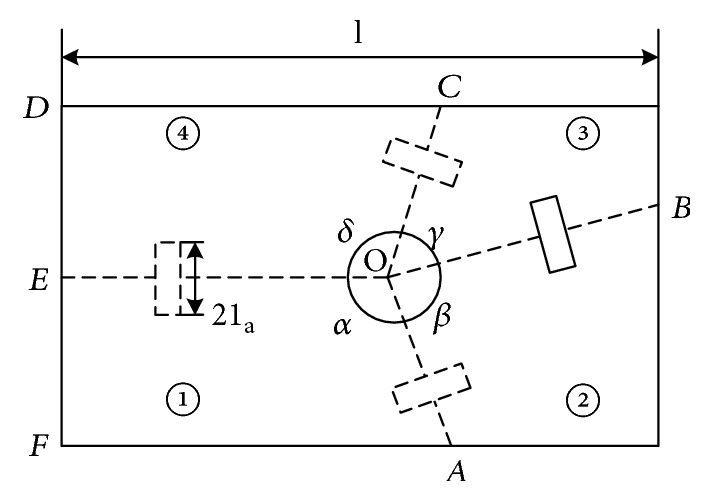
Structure of fully expanded wings.

**Figure 7:**
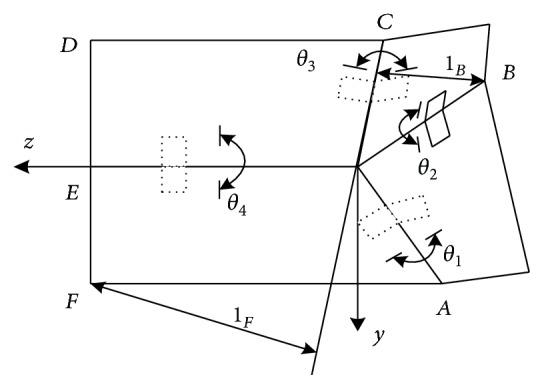
State of wings at *θ*_3_ = 135°.

Figure 6 shows the structure of the fully expanded wings.  Figure 7 is a schematic of the state of the wings at *θ*_3_ = 135°. In the folding movement, it is assumed that the center of mass of plate ②, plate ③, and their torsion hinges is at point *B*. Additionally, it is assumed that the center of mass of plate ① and its torsion hinges is at point *F*. Using these assumptions, the distance of *B* and *F* to *OC* can be calculated, respectively, using

(4) $$\begin{array}{c} l_{B}=\sin\gamma \frac{l-l_{OE}}{\cos\left(\pi -\gamma -\delta \right)},\\ l_{F}=\sqrt{{\left[l_{FE}\sin\left(\pi -\theta_{4}\right)\right]}^{2}+{\left[\sin\left(\delta -\frac{\pi }{2}\right)\left(\frac{l_{OE}}{\tan\left(\delta -\pi /2\right)}-l_{FE}\cos\left(\pi -\theta_{4}\right)\right)\right]}^{2}}. \end{array}$$

**Figure 8:**
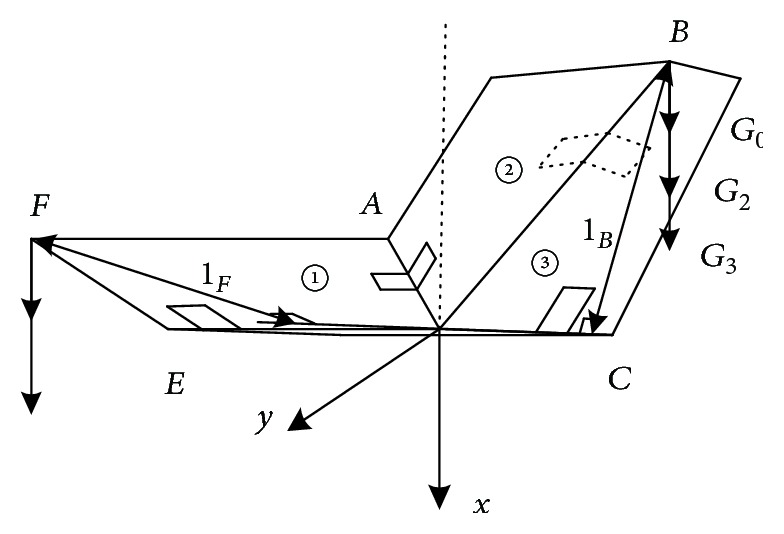
Diagram of stress analysis.

Figure 8 is a diagram of the stress analysis when the wings are in the state shown in Figure 7 (front view, clockwise deflection 45°). The torque of the plates and torsion hinges relative to the axis *OC* is calculated using

(5) $$\begin{array}{c} M_{1}=l_{F}\left(G_{1}+G_{0}\right)\cos\left(\pi -\theta_{4}\right), \end{array}$$

(6) $$\begin{array}{c} M_{2}=l_{B}\left(G_{2}+G_{0}\right)\cos\left(\pi -\theta_{3}\right), \end{array}$$

(7) $$\begin{array}{c} M_{3}=l_{B}\left(G_{3}+G_{0}\right)\cos\left(\pi -\theta_{3}\right), \end{array}$$

(8) $$\begin{array}{c} {Mf}_{1}={Mf}_{3}=f_{1}l_{a}+f_{1}l_{OA}\sin\left(\delta -\gamma \right), \end{array}$$

(9) $$\begin{array}{c} {Mf}_{2}={Mf}_{4}=f_{2}l_{OB}\sin\gamma +f_{2}\left[\frac{l_{OD}}{2}\sin\left(\pi -\delta \right)-l_{a}\cos\left(\pi -\delta \right)\right]. \end{array}$$

The mechanical analysis of the foldable wings was carried out using the software MATLAB. By analyzing the physical dimensions of the flexible wing of *Allomyrina dichotoma* and considering the output torque of the motor, the structural parameters of the bionic wing were determined, as shown in Table 2.

**Figure 9:**
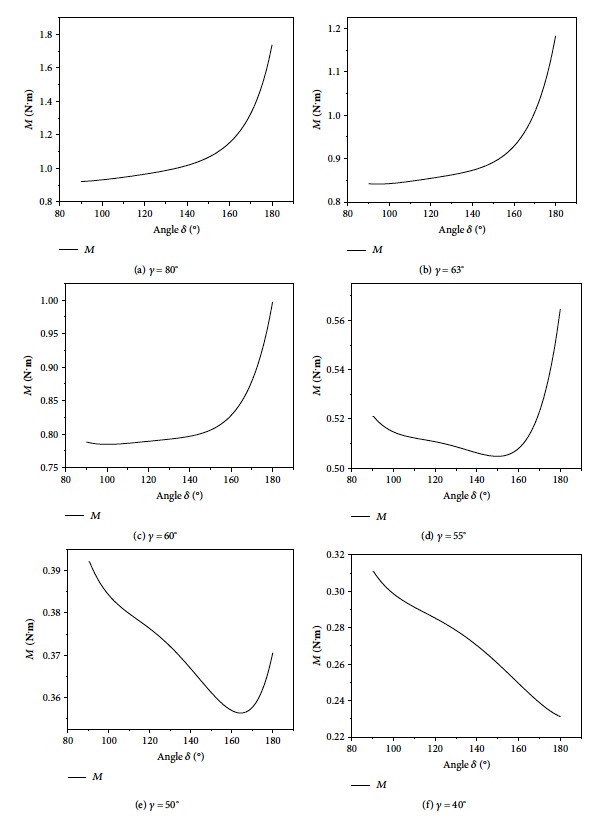
Output torques of the motor in different *γ* and *δ*.

The creasing angle of the plates greatly influences the output torque of the motor in the movement of wings. The initial output torque of the motor was obtained for different values of *γ* (80°, 63°, 60°, 55°, 50°, and 40°) and over a range of *δ*, as shown in Figure 9.

As shown in Figure 9, when *γ* > 63°, the curve has a continuous upward slope with increasing *δ*. When 40° < *γ* ≤ 63°, a local minimum in the output torque of the motor is observed. When *γ* = 40°, the output torque of the motor is at a minimum when *δ* = 180°. When *γ* is equal to 63°, 60°, 55°, 50°, and 40°, the minimum output torques of the motor are 0.842 N·m, 0.776 N·m, 0.504 N·m, 0.357 N·m, and 0.232 N·m, respectively.

**Figure 10:**
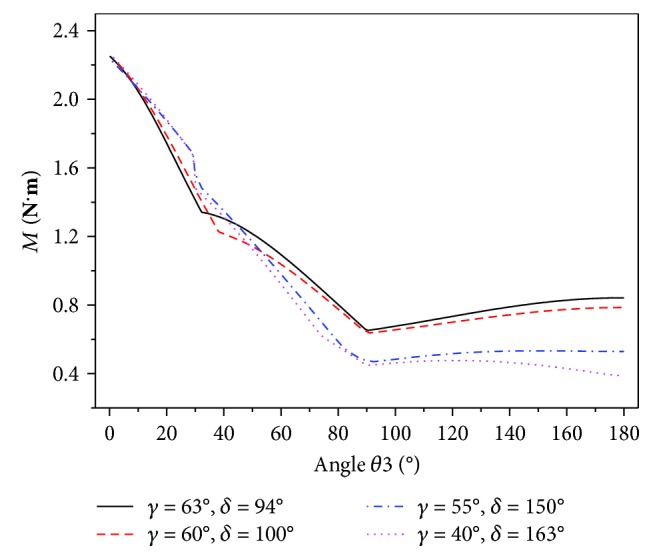
Output torque of the motor in different *γ*.

To compare the driven torque required for the wing movements at different *γ*, the output torque of the motor was simulated with a code implemented in MATLAB. The simulation results are shown in Figure 10. With the decreasing *γ*, the initial output torque of the motor also decreases. However, after the wings are completely folded, the final output torque of the motor remains unchanged at 2.264 N·m, since the output torque of the motor is primarily affected by the torque of the torsion hinges. The simulation results agree with the results expected in reality.

### 3.3. Analysis of the Folding Ratio of Wings

The folding ratio refers to the proportion of the existing area or volume to the original area or volume when an object is folded, which reflects the degree of folding. A higher folding ratio indicates a better folding effect. The static analysis model established in the present work ignores the effect of plate thickness, and when the wings are fully folded, the volumetric folding ratio is 100%. Analysis of the folding movement shows that the folding ratio is directly related to the creasing angle of the plates. According to the above analysis, the folding ratio of area for different plate creasing angles can be obtained.

1. When the angle *δ* = *π*/2 and *γ* = *π*/2, Fr = 75%.
2. When the angle *γ* < *π*/2 and *π*/2 < *δ* < *π*/2 + arctan((*l* − *l*_*OE*_)/*l*_*FE*_),

(10) $$\begin{array}{c} Fr=1-\frac{l_{OE}\cdot l_{FE}+\left(1/2\right){l_{OE}}^{2}\cdot \tan\left(\delta -\pi /2\right)+1/2{\left\{l-\left[l_{OE}+l_{FE}\cdot \tan\left(\delta -\left(\pi /2\right)\right)\right]\right\}}^{2}\cdot \tan\left(2\delta -\pi \right)}{l\cdot 2l_{FE}}. \end{array}$$

(3) When the angle *γ* < *π*/2 and (*π*/2) + arctan((*l* − *l*_*OE*_)/*l*_*FE*_) < *δ* < *π*,

(11) $$\begin{array}{c} Fr=1-\frac{l_{OE}\cdot l_{FE}+1/2\left(l-l_{OE}\right)\cdot \left[2l_{FE}-\left(l-l_{OE}\right)\cdot \tan\left(\pi -\delta \right)\right]}{l\cdot 2l_{FE}}. \end{array}$$

(4) When the angle *δ* = *π*, Fr = 50%.

**Figure 11:**
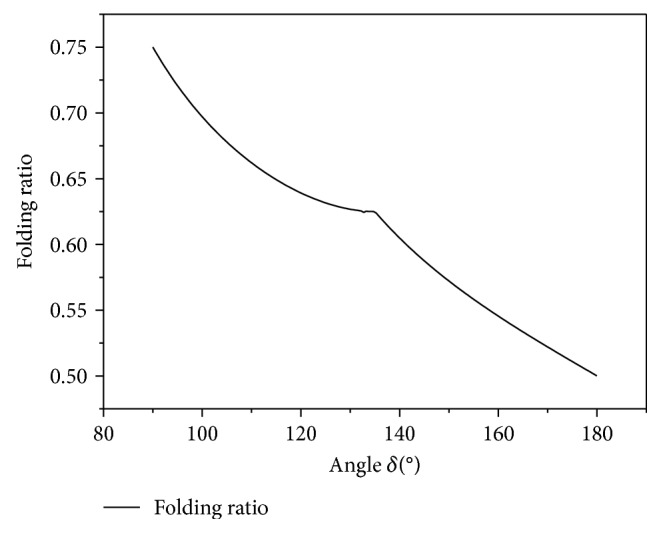
Folding ratio in different *δ*.

The curve of the folding ratio of wings as a function of *δ* is shown in Figure 11.

Through simulation analysis, it can be seen that when *γ* > 63°, it is impossible to calculate the effective minimum output torque of the motor. Therefore, the angles between the fold lines of the wings cannot be determined. The area folding ratio of wings cannot be found either. Using (10), the folding ratio of the area is calculated when *γ* is set to 63 °, 60 °, 55 °, 50 °, and 40 ° and when *δ* is 94 °, 100 °, 150 °, 163 °, and 180 °. The results are 72.5%, 69.7%, 57.1%, 53.7%, and 50%, respectively. Assuming that the output torque of the motor can be satisfied, the greater the wing folding ratio, the better the folding effect of the wing. Therefore, priority should be given to the wing folding ratio. Therefore, the creasing angles of the wing are set to *γ* = 63°, *δ* = 94°, *α* = 117°, and *β* = 96°.

**Figure 12:**
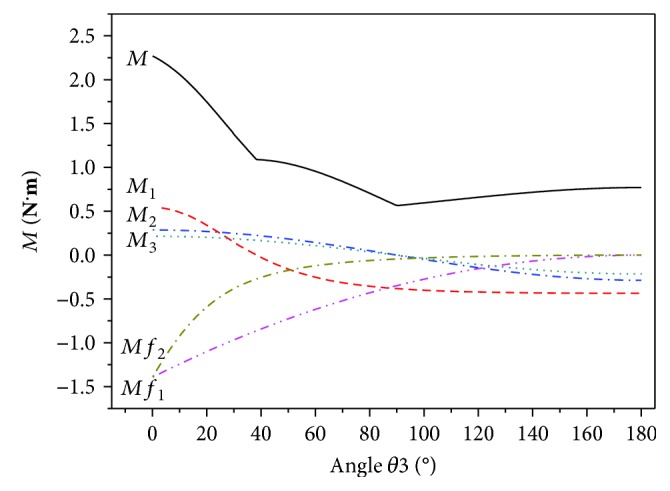
Torque of plates and torsion hinges.

The torque in the wing folding movement calculated by the MATLAB program is shown in Figure 12. The torque of each plate acting on the axis of rotation are *M*_1_, *M*_2_, and *M*_3_. Initially, the minimum output torque of the motor is *M* = 0.937 N·m. When 90° < *θ*_3_ < 180°, the output torque is reduced to 0.732 N·m, at which point *M* is mainly affected by the gravity of plates ② and ③. When 0° < *θ*_3_ < 90°, the output torque of the motor gradually increases to 2.264 N·m. The curve has an inflection point at *θ*_3_ = 40°. When 40° < *θ*_3_ < 90°, *M* is primarily affected by the gravity of plate ①. When 0° < *θ*_3_ < 40°, the bending deformation stress of torsion hinges are much greater than the gravity of the plates, and *M* is mainly affected by bending deformation of torsion hinges. As such, when 0° < *θ*_3_ < 40°, *M* increases faster. The curves of *Mf*_1_ and *Mf*_2_ in the figure show the moment of the elastic hinge with respect to the rotation axis. As the wings are folded, the deformation of the elastic hinge increases continuously and the force of the elastic hinge increases accordingly. As such, the resistance moment to the wing folding motion increases, which is expected.

## 4. Motion Simulation of Folding Wing

A 3D model of the bionic wing was established using SolidWorks software. The model was imported into ADAMS, and constraints and material properties were added. The driven functions based on the static analysis and the desired folding motion were also applied. The simulation type and step size and contact parameters based on known material properties were also set.

For the folding motion of bionic wings, the flexible deformation characteristics of the wings must be taken into account. In the present work, the model was processed with flexibility using ADAMS. The uniform velocities in the folding/unfolding movements were compared for two types of wings.

**Figure 13:**
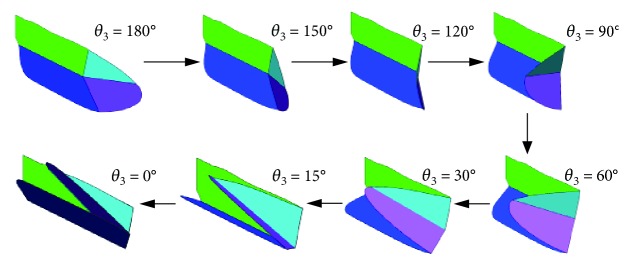
Folding movement of wings.

The simulation of the wing folding movement is shown in Figure 13. The driven force acts on the axis of rotation between plates ④ and ③, so that plate ③ moves toward plate ④. As the plates are all connected by torsion hinges, the rest of the plates are driven by the motion of plate ③, ultimately achieving the folding movement.

**Figure 14:**
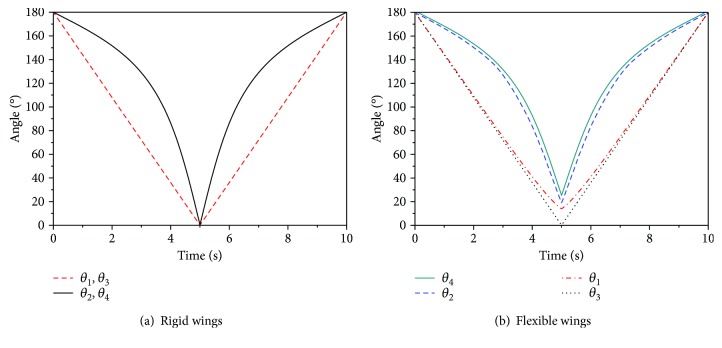
Dihedral angle of wings in the folding/unfolding motion.

After the simulation, the movement parameters of the wings under different conditions can be measured using the ADAMS postprocessor.  Figure 14(a) shows the dihedral angle of the rigid wings.  Figure 14(b) shows the dihedral angle of the flexible wings. The maximum deviation of the dihedral angle of the two types of wings is shown in Table 3. From Figures 5 and 14(a), it can be seen that the dihedral angle obtained by the kinematic mathematical model is consistent with the result of ADAMS simulation. The trends of the curves are both first slow and then fast. The observed inflection point is found at 131° and 133°, respectively. The inflection point error between the two methods is 1.53%. Compared with rigid wings, the flexible wings cannot be completely folded. The maximum deviation of the dihedral angle of the two types of wings is Δ*θ*_4_ = 24.4°, Δ*θ*_2_ = 19.1°, Δ*θ*_1_ = 14.2°, and Δ*θ*_3_ = 0°. The corresponding deviation ratios are 13.5%, 10.6%, 7.9%, and 0, respectively. From Table 3 and the kinematic model, it can be seen that when the wings are in the folded state, plate ① and plate ④ are on the outside in the folded direction and the dihedral angle between plates ① and ④ should be the largest. Therefore, Δ*θ*_4_ has a greater impact on the folding ratio of the wings. The smaller the value of Δ*θ*_4_, the greater the volumetric folding ratio of the wings.

**Figure 15:**
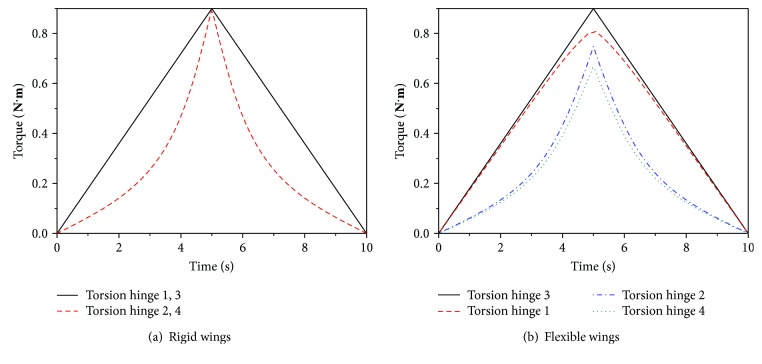
Torque of torsion hinges of wings.

The torque of the torsion hinges of the wings in the folding/unfolding motion was obtained from the simulation, as shown in Figure 15. The deviation in the maximum torque of the torsion hinges for the two types of wings is shown in Table 4. The maximum torque is *Mf* = 0.539 N·m. The deviations in the torque of torsion hinges of the two types of wings are Δ*Mf*_3_ = 0 N·m, Δ*Mf*_1_ = 0.14 N·m, Δ*Mf*_2_ = 0.26 N·m, and Δ*Mf*_4_ = 0.3 N·m. The deviation ratios are 0, 9.3%, 17.2%, and 19.9%, respectively. From Table 4 and the analysis of statics, we can obtain the stiffness and other physical parameters of torsion hinges that affect the output torque of the motor. The smaller the stiffness of the torsion hinges, the smaller the maximum output torque of motor.

## 5. Conclusion

1. A calculation model of the folding ratio for folding wing is established in this paper. According to the analysis of the wing folding ratio, the creasing angles of the plates of the bionic foldable wings are *γ* = 63°, *δ* = 94°, *α* = 117°, and *β* = 96°.
2. According to the kinematics of the bionic foldable wings, the dihedral angles between each fin plate are calculated. The results are compared with ADAMS dynamic simulation data, and the inflection point error between the two is 1.53%. This illustrates that the theoretical calculation is consistent with the simulation. Additionally, it proves that the design of the folding mechanism is reasonable.
3. The output torque of the motor is obtained by mechanics calculation and simulation. It shows that the smaller the stiffness of the torsion hinges, the smaller the maximum output torque of motor.
4. The results from simulating two types of wings show that the folding ratio of flexible wings in the fully folded state is less than that of the rigid wings. From the simulation and analysis, it was found that Δ*θ*_4_ has a greater impact on the folding ratio of wings. The smaller the value of Δ*θ*_4_, the greater the volumetric folding ratio of the wings. It is possible that in addition to the main motor, a motor could be added on the revolute pair between plates ① and ④. At the end of the folding process, the additional motor would drive plate ① to move closer to plate ④, which would achieve the purpose of reducing Δ*θ*_4_ and subsequently increase the folding ratio of the wings. It provides a basis for optimizing the design of the parameters of the folding wing.

## Figures and Tables

**Table 1:** D-H parameter of plates.

*i*	*a*_*i*−1_	*α*_*i*−1_	*d*_*i*_	*θ*_*i*_
1	0	*α*	0	*θ*_1_
2	0	*β*	0	*θ*_2_
3	0	*γ*	0	*θ*_3_
4	0	*δ*	0	*θ*_4_

**Table 2:** The parameters of wings.

Parameter name	Symbol	Value
Total length	*L*/mm	60
Total width	2*L*_*FE*_/mm	40
Length of OE	*L*_*OE*_/mm	32
Length of hinges	2*L*_*a*_/mm	14
Gravity of plates	*G*/N	0.2
Gravity of hinges	*G*_0_/N	5 × 10^−2^

**Table 3:** The maximum deviation of the dihedral angle.

	Δ*θ*_4_	Δ*θ*_2_	Δ*θ*_1_	Δ*θ*_3_
The deviation of the dihedral angle	24.4°	19.1°	14.2°	0°

**Table 4:** The difference in the maximum torque of the two types of wings.

	Δ*Mf*_4_	Δ*Mf*_1_	Δ*Mf*_2_	Δ*Mf*_3_
The difference of the torque	0.182 N·m	0.087 N·m	0.160 N·m	0 N·m

## Data Availability

The data used to support the findings of this study are available from the corresponding author upon request.
